# Intraoral Potentially Malignant Disorders in a Brazilian Oral Pathology Service: Epidemiological, Clinical, and Histopathological Findings

**DOI:** 10.1155/2018/2325808

**Published:** 2018-03-01

**Authors:** Fernanda Weber Mello, Gilberto Melo, Maria Inês Meurer, Elena Riet Correa Rivero

**Affiliations:** ^1^Postgraduate Program in Dentistry, Federal University of Santa Catarina, Florianópolis, SC, Brazil; ^2^Department of Pathology, Federal University of Santa Catarina, Florianópolis, SC, Brazil

## Abstract

The aim of this study was to investigate the characteristics of individuals with intraoral potentially malignant disorders (IOPMD) in an oral pathology service in Brazil. Cases were screened based on clinical diagnosis of leukoplakia (LKP), erythroleukoplakia (ELKP), and erythroplakia (EP). Clinical data and information regarding associated factors were gathered from biopsy reports. Histological diagnoses were collected from histopathological records. Among 208 IOPMD cases, 84.13% involved LKP; 11.1%, ELKP; and 4.8%, EP. The most affected sites were the gingiva and buccal mucosa. Histologically, epithelial dysplasia was present in 66.8% of the lesions, acanthosis and hyperkeratosis without epithelial dysplasia were present in 27.9%, and squamous cell carcinoma was present in 2.9%. Most patients were males, fair-skinned, with mean age of 53.4 years. Chronic smokers represented 73% of subjects, of which 30% also consumed alcohol. Smokers and drinkers were mostly males (*p* < 0.001). EP and ELKP represented histologically more severe degrees of epithelial dysplasia than LKP (*p* < 0.001). In conclusion, individuals with IOPMD were more frequently fair-skinned men in the sixth decade of life, with smoking habit. Special attention is required to clinical diagnoses of ELKP and EP since the prevalence of severe epithelial dysplasia, in situ carcinoma, and squamous cell carcinoma is higher than in LKP.

## 1. Introduction

The most frequent malignant neoplasm in the oral cavity is the oral squamous cell carcinoma (OSCC), a multifactorial disease in which smoked and/or smokeless tobacco is the main associated etiological factor [1, 2]. OSCC etiology varies worldwide; in Asian populations, the use of smokeless tobacco is highly associated with the development of OSCC [2]. On the other hand, in Brazil, the use of smokeless tobacco is rare and, therefore, the main etiological factor associated with OSCC development is consumption of the smoked form of tobacco [3, 4].

Clinically, intraoral potentially malignant disorders (IOPMD), such as leukoplakia (LKP), erythroplakia (EP), or mixed red and white lesions (erythroleukoplakia (ELKP) or speckled LKP), may precede the OSCC [5]. The diagnosis of IOPMD is based on clinical and histopathological characteristics. The clinical characteristics of LKP in particular may be misleading; therefore, clinicians must be able to rule out other oral white patches [6]. Histologically, these lesions can present some kind of epithelial alterations, such as epithelial dysplasia, hyperplasia, or in situ carcinoma (ISC); thus, biopsy and histopathological evaluation should be considered [7, 8]. More severe degrees of epithelial dysplasia, in which the epithelium is not organized in layers and presents with intense cellular atypia, are usually observed in red lesions, such as ELKP and EP, and in comparison with LKP, these lesions are most likely to be histologically diagnosed as in situ or invasive carcinomas [9].

In western countries, patients with IOPMD are usually fair-skinned males, around the fifth and sixth decades of life, with a history of chronic consumption of cigarettes and/or alcohol [10, 11]. The association with alcohol consumption significantly increases the risk of developing IOPMD and OSCC [10]. Published data for the Brazilian population showed that consumption of both smoked tobacco and alcohol increases the risk of developing OSCC by almost 10 times (OR = 9.65; 95% CI 1.57–59.08) [12]. Another possible risk factor associated with OSCC and IOPMD occurrence is infection with certain types of Human Papillomavirus (HPV), especially 16 and 18 [13, 14]. A recently published systematic review estimated that HPV infection was more associated with oropharynx/tonsils (38.29%) and tongue (20.34%) OSCC [15]. Furthermore, HPV infection is more frequently associated with OSCC in the posterior third of the tongue than in the anterior two-thirds [16].

The prevalence rates of IOPMD, clinical characteristics of patients and lesions, and etiological factors differ according to geographic location [10, 17, 18]. Regional differences could potentially affect the prevalence of IOPMD, justifying the importance of surveying the profiles of the lesions and affected patients [19]. Therefore, the aim of this study was to investigate the prevalence of the main types of IOPMD in a South Brazilian Oral Pathology Service, in order to identify the clinical characteristics of patients, associated etiological factors, and respective histological diagnosis of these lesions.

## 2. Material and Methods

After approval by the Ethics Committee of the authors' institution (protocol number 1.097.375), data were collected from registry files of the Oral Pathology Laboratory at the Federal University of Santa Catarina, Brazil, from March 2007 to October 2016. Intraoral cases clinically diagnosed as LKP, ELKP, or EP were selected. From these, only cases with histological diagnosis of epithelial acanthosis and hyperkeratosis without epithelial dysplasia (HKA), epithelial dysplasia (mild epithelial dysplasia (MiED), moderate epithelial dysplasia (MoED), and severe epithelial dysplasia (SED)), ISC, or OSCC were included in the sample. Cases with clinical suspicion for HPV infection were excluded from the sample.

Data regarding clinical diagnosis, gender, age, skin color of the patients, lesion anatomical site, and smoking and/or alcohol consumption habits were collected from biopsy reports. All biopsy material was prepared, stained with hematoxylin and eosin, and analyzed by the laboratory's oral pathology team, and pertinent information was registered in histopathological records, from which data regarding histological diagnoses were collected. Since data were collected from laboratory files, information about calibration among pathologists was not available. All cases were classified according to the World Health Organization (WHO) criteria, which classifies the degrees of epithelial dysplasia as “mild,” “moderate,” and “severe” [20].

Data were tabulated on Excel 2016 (Microsoft Office 2016, Microsoft) and analyzed using the statistical software SPSS Statistics 21 (IBM Corp., Armonk, NY, USA). A two-way Chi-square test and Fisher exact test were used to analyze associations between variables of interest (age, clinical and histological diagnoses, gender, and lesion anatomical site). The statistical significance was set at *α* = 0.05. Due to the limited number of included cases, data on clinical diagnosis were grouped into (1) LKP and (2) ELKP and EP. Data on histopathological diagnosis were grouped into (1) HKA, MiED, and MoED, and (2) SED, ISCC, and OSCC.

## 3. Results

The sample was composed of 208 cases (corresponding to 7.9% of the records in the laboratory file) from 137 individuals, from which 18 did not provide data on ethnicity, seven on patient age, and 23 about possible associated factor (e.g., smoked tobacco and alcohol consumption). The majority of individuals were males (56.9%) and fair-skinned (73.7%), with a mean age of 53.4 years. With regard to the associated etiological factors, 73% of the individuals were smokers, 23.4% were chronic users of alcohol, and 30% of the smokers self-reported concomitant chronic alcohol consumption (Table 1). An association was noted between gender and smoking and alcohol consumption (*p* < 0.001). The prevalence of both smoking and alcohol consumption habits was higher in males.

Although the characteristics of individuals were similar among those diagnosed with LKP, ELKP, and EP, some differences need to be highlighted. The male/female proportion was higher in the LKP and EP groups (1.27 : 1 and 2.33 : 1, resp.) than in the ELKP group (0.64 : 1). The prevalence of smoking habits was higher in individuals with LKP (76%) than in those with ELKP (56.52%) and EP (60%). Due to the absence of information regarding alcohol consumption and ethnicity in some biopsy reports (*n* = 141 and *n* = 27), these factors could not be appropriately analyzed in the different lesion groups.

With regard to clinical diagnosis, LKP was the most prevalent lesion, with its preferred locations being the gingiva and buccal mucosa (Table 2). Considering the final diagnosis, most cases of OSCC and ISC were diagnosed in the tongue, while HKA and MiED were more frequently observed in the gingiva and buccal mucosa (Table 3).

Individuals younger than 40 years constituted 18.2% of this sample. Most of these individuals were diagnosed with LKP (88%), and all of their lesions were histologically diagnosed as HKA, MiED, and MoED. All cases of SED, ISCC, and OSCC were diagnosed in patients older than 40 years and were most often clinically diagnosed with ELKP or EP.

**Figure 1:**
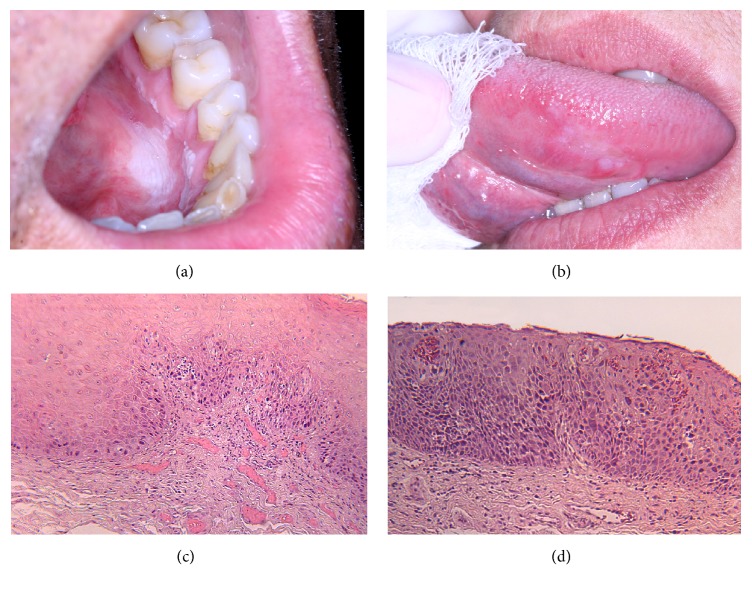
Clinical and histological correlations in two cases from the sample: (a) a lesion clinically described as oral leukoplakia involving the mouth floor and gingiva in the inferior left lingual mucosa; (b) a mixed red and white lesion, clinically described as erythroleukoplakia at ventral surface of the tongue; (c) histopathological mild epithelial dysplasia, with the epithelial layer showing acanthosis and cellular atypia restricted to the basal layer (H&E 400x); (d) histopathological severe epithelial dysplasia, with cellular atypia involving all layers of the epithelium (H&E 400x).

Table 4 shows that the prevalence of SED, ISCC, and OSCC increased from LKP to EP. An association was found between the clinical and histopathological diagnosis subgroups (*p* < 0.001). LKP was more frequently diagnosed with HKA, MiED, and MoED (Figures 1(a) and 1(c)), and ELKP and EP were more frequently diagnosed with SED, ISCC, and OSCC (Figures 1(b) and 1(d)).

## 4. Discussion

The prevalence of squamous cell carcinoma in lesions clinically diagnosed as IOPMD was 2.9%, which is not in agreement with a report from Brazil northeast region, in which this rate was considerably higher [21]. Moreover, the high prevalence of HKA observed in our sample should be highlighted; due to this study's design, frictional keratosis was not ruled out, which is a possible limitation. However, the occurrence of epithelial dysplasia among the cases in the laboratory file was similar to the results of previous studies [21, 22].

Normally, IOPMD patients are fair-skinned males in the fifth or sixth decade of life [23, 24], which is in accordance with this study sample characteristics. A possible explanation for this is that males are more exposed to the main etiological factors associated with OSCC, such as smoking and alcohol consumption. In addition, females usually attend to routine medical care more often, which might facilitate early diagnosis of these lesions [25]. In this sample, males were more exposed to smoking and alcohol consumption habits; however, no association was found between gender and histopathological diagnosis of the lesions, which might suggest that females could be exposed to other predictor factors.

In the Brazilian scenario, smoking is considered the main risk factor for oral cancer, particularly when associated with alcohol consumption habits [11, 12, 21]. In this study, it was observed that most of the sample was composed of smokers, and approximately 23% of individuals reported chronic alcohol consumption habit. Therefore, it is highly recommended to instruct these patients about the risks inherent to these habits, since the awareness of this condition may lead to an early diagnosis and prevent further complications [26]. It is important to develop campaigns to alert the population about the main risk factors associated with the development of IOPMD and OSCC, as well as the ways to prevent such health disorders. A recent study [27] on the association between IOPMD diagnoses and the quality of life of the patient showed an association between the diagnosis and functional and physical limitations, as well as with the psychological and social aspects of these individuals' lives. Therefore, health care professionals need to be vigilant, since diagnosing these lesions at earlier stages can provide a better prognosis and less treatment-related consequences for these individuals.

Typically, OSCC and IOPMD are noted in elderly individuals. However, over the past years, there has been an increase in the number of patients younger than 40 years diagnosed with OSCC [28]. In this sample, the majority of individuals under 40 years of age were diagnosed with LKPL; on the other hand, all cases of SED, ISC, and OSCC were diagnosed in individuals older than 40 years. The higher frequency of severe degrees of epithelial dysplasia found in the older group may have been influenced by the prevalence of smoking and alcohol consumption habits in this group.

Regarding IOPMD anatomical sites, the gingiva, followed by the buccal mucosa and tongue, was the most affected. This finding is inconsistent with most of data published by other authors, since the tongue, mouth floor, and gingiva were described as the preferred sites for IOPMD [5, 29]. One study from South India reported that the buccal mucosa was the most affected site by IOPMD [30]. It should be noted that, in Asian countries, there is a considerably high prevalence of oral cancer and IOPMD in this anatomical location, which is associated with the consumption of smokeless tobacco (areca nut/betel quid) that is usually applied to the buccal mucosa several times a day [31]. In western countries, the etiological factor showing greatest association is the consumption of smoked tobacco [32]. Therefore, the variability of etiological factors worldwide must be taken into account when analyzing the epidemiology of IOPMD in different geographic locations.

Another possible confounding factor regarding this study population is HPV related IOPMD. However, in this sample HPV screening was performed based on clinical suspicion, and no laboratory exams, such as polymerase chain reaction (PCR), were performed to confirm diagnosis; thus, these lesions could not be completely ruled out. Some previous studies reported that, in Brazil, the prevalence of HPV infection (types 16 and 18) in oral cancer is lower than in other countries [33–35]. The global prevalence of HPV was reported to be higher in oral potentially malignant disorders (OPMD) cases than in controls (OR: 3.98, 95% CI: 2.62–6.02), and among the different subgroups of OPMD, the prevalence was higher in the dysplasia (OR: 5.10, 95% CI: 2.03–12.80) and leukoplakia (OR: 4.03, 95% CI: 2.34–6.92) subgroups [36]. Additionally, it is important to emphasize that HPV related cancers are more prevalent in oropharynx, including tonsils and the posterior third of the tongue, than in other intraoral subsites [16, 37, 38].

There were a high prevalence of HKA in the buccal mucosa and considerably lower rates of SED, ISCC, and OSCC in this location. The occurrence of these lesions may be influenced by occlusal trauma at the buccal mucosa, resulting in reactive keratosis, which could be clinically misdiagnosed as LKP [6]. It is important to consider that some authors suggested that red lesions present higher rates of undergoing malignant transformation than white ones [9]. In the sample findings from this study, ELKP and EP were significantly associated with more severe degrees of epithelial dysplasia than LKP, which is in accordance with reports from previous studies [21, 39]. Therefore, the need for performing biopsy in these lesions is highlighted.

The majority of our sample was clinically diagnosed as LKP, while histologically most of these lesions presented some degree of epithelial dysplasia. This finding differs from the literature, in which the majority of the lesions clinically diagnosed as LKP were histologically reported as hyperkeratosis without epithelial dysplasia [21, 40]. This result may be related to the larger smoker group in our sample in comparison with other studies [11, 41], which could have influenced the high prevalence of epithelial dysplasia. In addition, some authors suggested that, in white lesions with possible mechanical irritants, a clinical follow-up should be performed in order to eliminate possible etiological factors, and biopsy should only be performed when the remission of white lesions is not observed [42]. This type of management could also have influenced the prevalence of epithelial dysplasia in this sample. Another important fact is that the criteria used for diagnosing the degree of epithelial dysplasia are subjective and are poorly reproducible between examiners, which may lead to different histological interpretations of these lesions [43, 44].

This study had some limitations, since clinical information about patients was not directly collected by the authors, and the available data were dependent on the correct filling of the biopsy reports by the professional who performed the biopsy procedure. Likewise, data concerning smoking and alcohol consumption depended on the patient self-report. Under these circumstances, we would like to highlight the risk of bias inherent to this study design.

## 5. Conclusions

The results of the present study suggest that the majority of individuals with IOPMD were fair-skinned males in the sixth decade of life, with a chronic smoking habit being the most frequently reported factor. Alcohol consumption and smoking habits were more frequent in males than females. Clinical diagnoses of ELKP and EP were associated with a higher prevalence of SED, ISC, and OSCC when compared to LKP. Due to the higher prevalence of SED, ISC, and OSCC in the tongue, special attention should be given when IOPMD are located on the ventral surface or lateral border of the tongue.

## Figures and Tables

**Table 1:** Comparison between clinical and histopathological diagnosis.

Clinical characteristics	Male (*n* = 78)	Female (*n* = 59)	Total (*n* = 137)
Age (mean ± SD)	53 ± 9.5	53.9 ± 9.6	53.4 ± 9.5
Smoking (*n*, %)	65 (83.3)	35 (59.3)	100 (73)
Alcohol consumption (*n*, %)	29 (37.2)	3 (5.1)	32 (23.4)
Smoking and alcohol consumption (*n*, %)	28 (35.9)	2 (3.4)	30 (21.9)

SD, standard deviation.

**Table 2:** Location of the lesions according to the clinical diagnosis.

	Clinical diagnosis, *n* (%)			
	LKP (*n* = 175)	ELKP (*n* = 23)	EP (*n* = 10)	Total (*n* = 208)
Mouth floor	16 (9.1)	2 (8.7)	-	18 (8.7)
Tongue	31 (17.7)	7 (30.4)	3 (30.0)	41 (19.7)
Gingiva	53 (30.3)	4 (17.4)	2 (20.0)	59 (28.4)
Buccal mucosa	44 (25.1)	8 (34.8)	4 (40.0)	56 (26.9)
Palate	12 (6.9)	2 (8.7)	1 (10.0)	15 (7.2)
Retro molar	19 (10.9)	-	-	19 (9.1)

LKP, leukoplakia; ELKP, erythroleukoplakia; EP, erythroplakia.

**Table 3:** Location of the lesions according to the histopathological diagnosis.

	Histopathological diagnosis, *n* (%)						
	HKA (*n* = 58)	MiED (*n* = 85)	MoED (*n* = 40)	SED (*n* = 14)	ISC (*n* = 5)	OSCC (*n* = 6)	Total (*n* = 208)
Mouth floor	2 (3.5)	8 (9.4)	3 (7.5)	4 (28.6)	-	1 (16.7)	18 (8.7)
Tongue	8 (13.8)	13 (15.3)	9 (22.5)	3 (21.4)	5 (100)	3 (50.0)	41 (19.7)
Gingiva	14 (24.1)	29 (34.1)	14 (35.0)	1 (7.1)	-	1 (16.7)	59 (28.4)
Buccal mucosa	23 (39.7)	19 (22.3)	8 (20.0)	6 (42.9)	-	-	56 (26.9)
Palate	5 (8.6)	6 (7.1)	3 (7.5)	-	-	1 (16.7)	15 (7.2)
Retro molar	6 (10.3)	10 (11.8)	3 (7.5)	-	-	-	19 (9.1)

HKA, hyperkeratosis and acanthosis without epithelial dysplasia; MiED, mild epithelial dysplasia; MoED, moderate epithelial dysplasia; SED, severe epithelial dysplasia; ISC, in situ carcinoma; OSCC, oral squamous cell carcinoma.

**Table 4:** Comparison between clinical and histopathological diagnosis.

Histopathological diagnosis	Clinical diagnosis, *n* (%)			
	LKP (*n* = 175)	ELKP (*n* = 23)	EP (*n* = 10)	Total (*n* = 208)
HKA	58 (33.1)	-	-	58 (27.9)
MiED	76 (43.4)	8 (34.8)	1 (10.0)	85 (40.9)
MoED	31 (17.7)	7 (30.4)	2 (20.0)	40 (19.2)
SED	5 (2.9)	5 (21.7)	4 (40.0)	14 (6.7)
ISC	2 (1.1)	1 (4.4)	2 (20.0)	5 (2.4)
OSCC	3 (1.8)	2 (8.7)	1 (10.0)	6 (2.9)

HKA, hyperkeratosis and acanthosis without epithelial dysplasia; MiED, mild epithelial dysplasia; MoED, moderate epithelial dysplasia; SED, severe epithelial dysplasia; ISC, in situ carcinoma; OSCC, oral squamous cell carcinoma; LKP, leukoplakia; ELKP, erythroleukoplakia; EP, erythroplakia.
